# Supplementary material for: Time-series analysis for forecasting monthly workload at two elephant hospitals in Thailand
Source: PLoS One. 2025 Dec 30;20(12):e0337825. doi: 10.1371/journal.pone.0337825 (PMC12752977; doi:10.1371/journal.pone.0337825)
Supplement: S1 Fig — NEI showed sustained positive autocorrelation across short lags, consistent with underlying seasonal or cyclical workload patterns. In contrast, DLD displayed weak or no autocorrelation, indicating stochastic or irregular admission dynamics. (PDF) [file pone.0337825.s001.pdf]

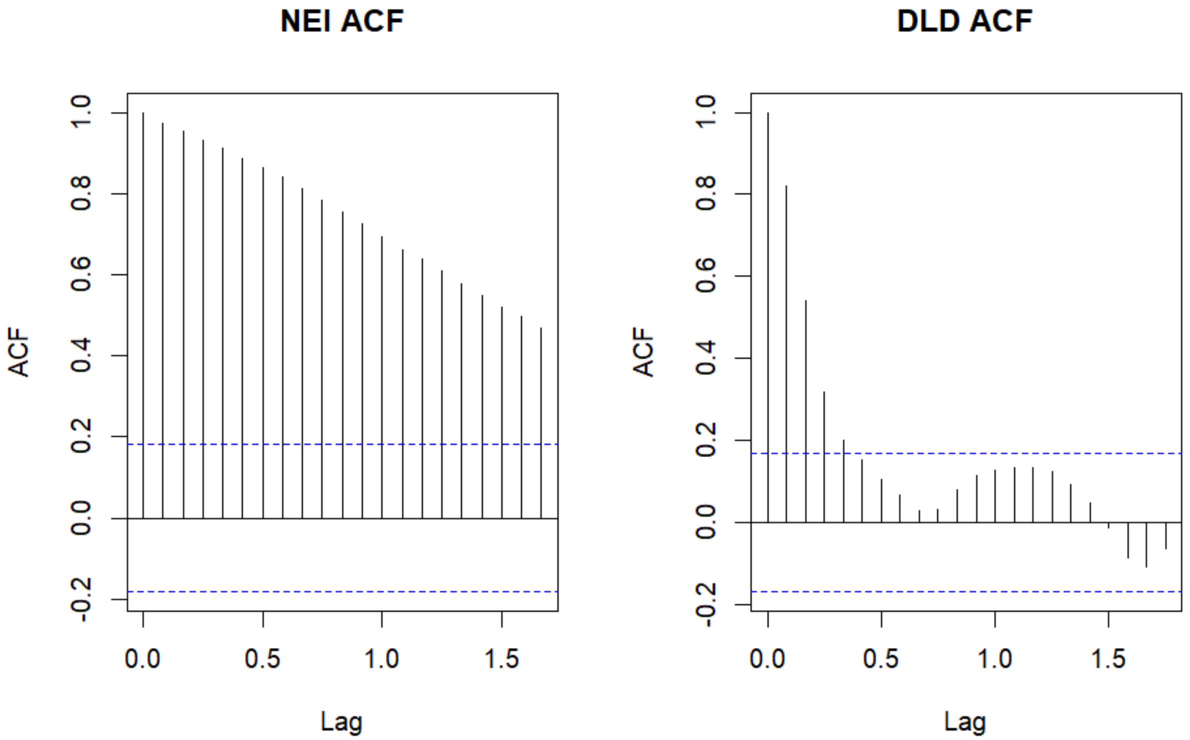

S1 Fig. Autocorrelation function (ACF) plots of monthly active caseloads at NEI (left) and DLD (right) elephant hospitals. NEI showed sustained positive autocorrelation across short lags, consistent with underlying seasonal or cyclical workload patterns. In contrast, DLD displayed weak or no autocorrelation, indicating stochastic or irregular admission dynamics.
