# Supplementary material for: Time-series analysis for forecasting monthly workload at two elephant hospitals in Thailand
Source: PLoS One. 2025 Dec 30;20(12):e0337825. doi: 10.1371/journal.pone.0337825 (PMC12752977; doi:10.1371/journal.pone.0337825)
Supplement: S1 Table — (PDF) [file pone.0337825.s002.pdf]

S1 Table. Statistical comparison between NEI and DLD hospital datasets.

Metric	NEI (mean ± SD)	DLD (mean ± SD)	Test used	Statistic	P-value
Age (years)	33.66 ± 21.66	22.60 ± 18.63	Welch's t-test	t = -6.50 (df = 396.7)	<0.001***
			Wilcoxon rank-sum	W = 3 = 30544	<0.001***
Length of stay (days)	92.41 ± 286.99	177.50 ± 378.45	Welch's t-test	t = 1.57 (df = 57.7)	0.121
			Wilcoxon rank-sum	W = 19715	<0.001***
Monthly caseload variance	-	-	Levene's test	F = 282.4 (df = 1246)	<0.001***

NEI = National Elephant Institute hospital; DLD = Department of Livestock Development hospital

*** indicated a significant difference at P<0.01
