# Supplementary material for: Time-series analysis for forecasting monthly workload at two elephant hospitals in Thailand
Source: PLoS One. 2025 Dec 30;20(12):e0337825. doi: 10.1371/journal.pone.0337825 (PMC12752977; doi:10.1371/journal.pone.0337825)
Supplement: Attachment — Submitted filename: Response to Reviewers.pdf [file pone.0337825.s003.pdf]

General Response

We would like to thank the Academic Editor and both reviewers for their valuable feedback, which has helped us significantly improve the clarity, structure, and scientific rigor of the manuscript. We have carefully revised the manuscript in response to all comments and believe the current version is now substantially strengthened.

Summary of Key Revisions:

- Improved clarity, grammar, and logical flow throughout all sections.

- Reorganized the Methods and Discussion section with clear subheadings.

- Strengthened the justification for dataset selection and acknowledged limitations.

- Added Data Availability statement and evaluation metric definitions.

All changes are clearly marked in the revised manuscript with track changes for full transparency. Below is our detailed response to the Academic Editor and both reviewers. Changes made in the manuscript are described with reference to the cleaned version (i.e., line numbers refer to the tracked-clean manuscript). Reviewer comments are in **bold**; our responses are in red.

Additional Editor Comment:

While the study addresses an interesting topic, significant revisions are required. In particular, the manuscript's structure and clarity need improvement. Please ensure that the Methods, Results, and Discussion sections are clearly delineated and that language redundancies are removed. The study should also better acknowledge and justify its data limitations (e.g., small sample size and inconsistent years of data). Finally, include a Data Availability statement per PLOS ONE guidelines, ensuring that underlying data and code are shared.

Response:

We thank the Academic Editor for the constructive feedback, which greatly improved the manuscript. In response:

- **Structural clarity**: We restructured the manuscript to delineate Materials and Methods, Results, and Discussion more clearly. The Materials and Methods section is now organized with subheadings: Study sites and data sources, Forecasting workflow, Time-series decomposition and seasonality testing, Model descriptions, and Model evaluation and forecasting (Lines 103–230). Discussion is now organized into clear subsections, including Hospital-specific caseload patterns (Lines 353-381), Seasonal disease trends (Lines 382-422), Model interpretability (Lines 423-437), Forecast implications and systemic gaps (Lines 438-458), Limitations and future directions (Lines 459–478).
- **Language and readability**: We thoroughly edited the manuscript to remove redundant phrasing and improve clarity across all sections, particularly in the Introduction and Discussion.
- **Acknowledgment of data limitations**: The revised manuscript now explicitly explains that elephant hospitalizations are inherently rare, and that hospital records prior to 2016 (DLD) and 2020 (NEI) were largely paper-based and inconsistent. This constrained both sample size and time-series length. These limitations are acknowledged in:
    - Introduction (Lines 82–86, 94–98)
    - Methods (Lines 113–118)
    - Discussion (Lines 339–341, 347-352, 453-454, 466-467)
    - Conclusion (Lines 490-492)

- **Data availability compliance**: We have added a Data Availability Statement in accordance with PLOS ONE guidelines, indicating that raw data are available upon reasonable request due to privacy constraints, and that the full R code is shared via public repository (Lines 507–511).

We believe these revisions address all components of the editor's and reviewer's concerns and significantly enhance the clarity, rigor, and transparency of the manuscript.

Reviewer #1:

The provided article implementing several time-series estimation methods to forecast the workload of elephant hospitals. Although the author's justification of the novelty of the suggested idea and domain sounds reasonable, the article lacks the reasonable justification and explanation about the experimental results. Consequently, the provided article is not formatted to be an appropriate scientific article which is the reason why I suggest to reject the given paper.

Response:

While we acknowledge the reviewer's concern, we believe the revised version now provides clearer justification. To address this, we have thoroughly revised the manuscript as follows:

- The Materials and Methods section is now organized with subheadings: Study sites and data sources, Forecasting workflow, Time-series decomposition and seasonality testing, Model descriptions, and Model evaluation and forecasting (Lines 103–230).

We have added model justification in Lines (156-160):
"Six time-series models were employed to predict the monthly hospital workload at both study sites. These forecasting models were chosen to cover diverse analytical approaches. Classical statistical methods (ARIMA/SARIMA, ETS, TBATS) were selected to handle linear and seasonal patterns, while machine learning-based methods (NNTR, Prophet, XGBoost) were included to capture potential non-linear relationships and more complex data structures."

- The Discussion section is now organized into clear subsections: Hospital-specific caseload patterns (Lines 353-381), Seasonal disease trends (Lines 382-422), Model interpretability (Lines 423-437), Forecast implications and systemic gaps (Lines 438-458), Limitations and future directions (Lines 459–478)—to improve clarity and scientific presentation.
- Removed redundancy, and improved precision and clarification throughout.

These changes directly address the previous lack of explanation regarding experimental results. We now explicitly link model outputs to data structure, seasonal trends, hospital-level dynamics, and broader system-level challenges. We believe this revision now presents a cohesive scientific narrative with improved rigor and relevance to the field.

Q1. Is there significant difference exist in dataset of NEI and DLD? If there exists, the author should provide and justify such difference. If there exists no significant difference, the author should explain why all six model demonstrate significantly different performance in two distinct dataset in Table 1.

Response:

Thank you for this important observation. We agree that the difference in model performance warrants clearer justification. While seasonality testing was already included in the original manuscript (WO-test: NEI significant, DLD non-significant, Lines 262-267), we have now made this distinction more explicit and expanded our interpretation of how data structure influenced model performance.

- We have added Supplementary Table S1 and Fig S1 to highlight the structural differences between datasets. Table S1 confirms demographic and caseload variation (e.g., older elephants and more stable length of stay at NEI), while Fig S1 illustrates stronger seasonal autocorrelation at NEI and highly irregular patterns at DLD. These findings clarify why ETS outperformed at NEI and NNTR at DLD.
- Revised content added in the Materials and Methods at Lines 149-154:

"To further characterize the underlying structure of each dataset, statistical comparisons between NEI and DLD hospital data were conducted. Descriptive comparisons included age, length of stay (LOS), and active monthly caseloads. Welch's *t*-test and Wilcoxon rank-sum test were used for continuous variables (e.g., age, LOS), while variance differences in monthly caseloads were assessed using Levene's test. Additionally, autocorrelation patterns were explored using autocorrelation function (ACF) plots to assess temporal dependencies."

- We have added the Results compared between two hospital datasets at Lines 257-260:

"Descriptive and statistical comparisons between hospitals confirmed structural differences between datasets (Table S1). Elephants at NEI were significantly older and had shorter but more regular stays, while DLD exhibited highly variable monthly caseloads and minimal autocorrelation (Fig. S1), reflecting stochastic admission patterns."

- We have edited the Discussion for clarification:

"These hospitals exhibited contrasting patterns in disease prevalence, seasonality, and caseload dynamics. NEI showed consistent seasonal trends, best modeled by ETS, while DLD displayed irregular fluctuations, more accurately captured by NNTR. These

differences underscore the importance of tailoring forecasting approaches to the specific data structure of each setting." (Lines 343-347)

"Collectively, the contrasting model performances reflect fundamental differences in the patient demographics (e.g., more geriatric chronic cases at NEI), regional elephant management (tourism vs. private ownership), and healthcare-seeking behaviors." (Lines 378-381)

"Seasonal analysis showed a clear pattern in hospital admissions at NEI, but no distinct seasonality at DLD. ……. These divergent activity patterns may influence both the timing and nature of health issues requiring hospital care." (Lines 382–390)

- To further emphasize this point, we revised the Discussion to explain that NEI had more structured seasonal patterns and stable admission counts, while DLD had irregular fluctuations likely influenced by external factors, such as community ownership, regional access barriers, and pandemic-related elephant movements. These details were added on Lines 353-422 and expanded on Lines 439–452.

These additional tests confirmed the discrepancy between NEI and DLD. We believe this enhanced explanation now addresses the core of the reviewer's concern by linking model performance to the underlying temporal structure of the datasets.

Q2. Although the author suggests prediction results in NEI and DLD dataset, I cannot find the main claim of the author that shows the main contribution of the presented work. If it is natural to have different prediction results in different dataset, I declare that two distinct dataset is too small set to show the novelty of the work. If the author targeted to make the general forecasting methods, the generalization of the method in distinct dataset is absence. Therefore, I cannot find the main claim of the given paper to have the contribution in the domain.

Response:

We appreciate the reviewer's request for greater emphasis on the paper's central contribution. While the original manuscript briefly mentioned that our study demonstrates feasibility of predictive analytics in elephant hospitals, we agree that the framing needed to be strengthened.

We now clearly articulate the main contribution and novelty of the study in multiple sections:

- In the **Abstract**, we revised the final sentences to state:

"This study provides the first demonstration that multi-model time-series forecasting can generate actionable insights using sparse, real-world data from wildlife hospitals. By embedding predictive analytics into routine operations, it offers a novel, scalable framework to support data-driven decision-making in endangered species care." (Lines 36–39)

- In the **Introduction**, we strengthened the framing of the study's contribution as a first application of time-series forecasting to elephant hospitals, and a rare example in wildlife veterinary medicine. We added:

"Although elephant hospitals serve fewer patients than human facilities, the high per-patient cost and the species' conservation significance make proactive forecasting no less critical." (Lines 82–84)

and
"While elephant hospital datasets are inherently small due to the rarity of hospitalizations, this study demonstrates that robust forecasting remains feasible and informative. To our knowledge, this is the first study to systematically evaluate and compare multiple forecasting models using sparse, real-world data from wildlife hospitals, offering a novel tool for strategic care planning." (Lines 94–98)

- In the **Discussion**, we added:

"This study presents a novel application of predictive analytics in captive Asian elephant healthcare, demonstrating that it is both feasible and informative to forecast monthly hospital admissions using sparse, real-world datasets." (Lines 339-341) at the opening.

"Importantly, this study provides one of the first demonstrations that predictive forecasting frameworks, common in human health system, can be effectively adapted for wildlife hospitals that manage few patients but require disproportionately high resource use per case. By demonstrating that robust, model-driven forecasts are possible even with limited datasets, and can directly inform hospital-level decision-making in conservation settings. This approach offers tangible value in optimizing staffing, supply chains, and emergency preparedness in endangered species care." (Lines 347-352)

"Accurate forecasting in such divergent clinical settings has practical implications for veterinary resource planning, particularly in specialized, resource-limited elephant care facilities." (Lines 337-339)

"Importantly, even in resource-limited veterinary contexts, forecasting holds practical utility. Projections can inform medication procurement schedules, guide staffing needs

during peak periods, and support facility preparedness. The ability to shift from reactive to anticipatory logistics is especially valuable in endangered species care, where treatment delays can be fatal and resource shortages can jeopardize animal welfare." (Lines 453-458)

- In the **Conclusion**, we added:

"This study demonstrated that time-series forecasting, while traditionally applied in high-volume human health settings, can be effectively adapted to wildlife hospitals with low patient volume but high per-case resource demands." (Lines 481-484)

"Crucially, this study provides the first demonstration that meaningful, actionable forecasts can be generated from sparse, real-world datasets in endangered species care. These predictions offer operational value by informing staffing plans, guiding medical supply procurement, and strengthening emergency readiness. As such, this work serves as a first template for data-driven planning in wildlife hospitals, an area previously unsupported by forecasting science." (Lines 490-495)

"As the veterinary care of captive wildlife becomes increasingly complex, the integration of predictive analytics into hospital systems offers a scalable, conservation-aligned strategy to improve both animal welfare and healthcare system resilience." (Lines 500-503)

We acknowledged that our study does not aim to generalize forecasting methods universally, but to illustrate model selection tailored to hospital-specific dynamics. We emphasize that generalizability would require a larger, national dataset, which we now suggest as a next step (Lines 471-478).

We have added:

"…data availability differed between sites (five years for NEI, nine for DLD), limiting model comparability and trend detection. This discrepancy stem from reliance on paper-based records which introduced risks of missing or incomplete data and affecting historical accuracy." (Lines 463-466)

and

"…the sample size remains modest due to the rarity of elephant hospitalization, which may constraint model generalizability." (Line 466-467)

Q3. Please write the paper to be clear and sound reasonable.

Response:

We thank the reviewer for this important recommendation. In response, we conducted a comprehensive revision of the manuscript to improve clarity, logical flow, and scientific presentation. Specific improvements are summarized below, with corresponding line numbers from the revised manuscript:

- **Abstract**: Rewritten for clarity, conciseness, and emphasis on the study's central contribution. (Revised content: Lines 19–39)
- **Introduction**: Reorganized to flow logically from background context to problem statement and study rationale. Redundancy was removed and justification for forecasting in low-volume wildlife hospitals was strengthened. (Revised content: Lines 42-101)
- **Methods:** Fully restructured using clear subheadings to enhance navigability and transparency of study design, data processing, model development, and evaluation metrics. (Revised content: Lines 103-230)
- **Results**: Clarified comparisons across sites and models. Redundant phrasing was removed and statistical findings were presented with improved precision. (Revised content: Lines 232–331)
- **Discussion:** Structured into five subsections for clarity:
  (1) Hospital-specific caseload patterns (Lines 353–381)
  (2) Seasonal disease trends (Lines 382–422)
  (3) Model interpretability (Lines 423–437)
  (4) Forecast implications and systemic gaps (Lines 438–458)
  (5) Limitations and future directions (Lines 459–478)
  These revisions highlight the contrast between the two hospitals and clearly link model performance to hospital context, seasonality, and operational characteristics.
- **Conclusion:** Reframed to reiterate the practical contribution and recommendation for national-level implementation. (Lines 480–503)

We also addressed minor grammatical issues and improved academic tone throughout the manuscript. All track changes are visible in the Revised Manuscript with Track Changes for transparency.

Reviewer #2:

The article addresses an important and unique problem—adapting time series forecasting approaches used for human infectious diseases to veterinary medicine, namely, for elephants. The creation and analysis of a digital dataset from two hospitals in Thailand, specifically for elephants, is a valuable contribution. However, there are several critical areas that need attention:

Response to Reviewer #2

We are grateful for your thoughtful comments and recognition of the study's potential to contribute to veterinary analytics. Your suggestions prompted several critical improvements in our manuscript. We have revised the structure, clarified justifications, and enhanced technical definitions to ensure the manuscript is both accessible and scientifically rigorous. Below, we address each concern point by point and indicate where changes were made in the revised manuscript.

Q1. Justification of the Study:
While studying elephant health is undoubtedly valuable, particularly given their endangered status (as listed by the IUCN), the rationale for forecasting illness rates among such a small population needs further clarification. Is there a known or emerging infectious disease affecting elephants in Thailand that motivates this study? Given the limited number of existing elephants, the number of potentially sick individuals may be too low to justify complex forecasting.

Additionally, the normal operational capacity of the hospitals should be described to understand the practical implications of a potential increase in cases.

Response:

We agree and have revised both the **Abstract** and **Introduction** to better justify the use of forecasting tools in this low-volume but high-resource demand context. New contents have now added:

- In **Abstract**, we have added:

"Although patient volume was low, each case demanded disproportionately high resources, justifying the need for anticipatory planning." (Lines 34-36)

- **Introduction:** We now explicitly state that while the number of cases is relatively small compared to human hospitals, each case requires disproportionately high resources. This is particularly critical in managing resource-intensive diseases like EEHV-HD and severe colic, which have clear seasonal peaks and can overwhelm hospital operations.

"……These recurring, resource-intensive cases demand forward-looking systems to support continuous, high-quality care." (Lines 67-73)

"Although elephant hospitals serve fewer patients than human facilities, the high per-patient cost and the species' conservation significance make proactive forecasting no less critical. In addition, proactive forecasting is particularly relevant for recurring, high-cost conditions such as EEHV-HD and colic, commonly seen in Thai captive elephants, which often drive intense medical resource requirement." (Lines 82-86)

"While elephant hospital datasets are inherently small due to the rarity of hospitalizations, this study demonstrates that robust forecasting remains feasible and informative." (Lines 94–96)

These additions directly respond to the reviewer's concern by grounding the need for forecasting in disease burden, resource logistics, and seasonal surges, even within a small population.

We have clarified that both hospitals frequently operate near their admission limits during seasonal peaks:

- In the **Introduction**, we have added:

"During periods of high demand, they frequently operate near full capacity, underscoring the need for robust caseload forecasting to optimize staffing, treatment preparation, and resource allocation." (Lines 62–64)

- In the **Materials and Methods**, we have added

"NEI hospital, located in a forested area, can accommodate up to 20 elephants at a time. Its spacious, nature-integrated layout allows stable cases to be housed beyond the core hospital unit when needed. In contrast, DLD hospital is situated in a constrained urban area and can manage no more than three elephants concurrently." (Lines 109–112)

Q2. Ambiguity in Analysis:
The choice of time windows for the two hospitals is inconsistent—five years for one and nine for the other. A consistent time frame would allow for better comparison across models and datasets. The rationale behind this discrepancy should be clearly explained, or the data range should be standardized.

Response:

Thank you for this important point. We retained the five- and nine-year datasets due to the limitations in data availability but have now explicitly justified this decision. We have clarified our rationale in the Methods section, explaining that record reliability varies between hospitals. Prior to 2016 (DLD) and 2020 (NEI), records were mostly paper-based and incomplete, limiting comparability.

- In the **Methods** section, we have added:

"Historical data availability constrained our dataset selection… These study periods were chosen because record-keeping prior to 2016 (DLD) and 2020 (NEI) was predominantly paper-based and inconsistent; thus, we focused on the years with the most complete and reliable data for analysis." (Lines 113–118)

- Additionally, we acknowledged this as a **limitation**:

"….data availability differed between sites (five years for NEI, nine for DLD), limiting model comparability and trend detection. This discrepancy stem from reliance on paper-based records which introduced risks of missing or incomplete data and affecting historical accuracy. Third, the sample size remains modest due to the rarity of elephant hospitalization, which may constraint model generalizability." (Lines 463–467)

These explanations aim to transparently address the discrepancy while maintaining the study's integrity based on available evidence.

Q3. Paper Structure:
The manuscript would benefit from a major structural revision. Sectioning is insufficient, and content is often presented in inappropriate sections. For instance, the methodological pipeline is briefly introduced in the Data section (lines 128–132),

which is not ideal. A clearer separation between data description, methodology, results, and discussion would greatly improve readability and comprehension.

Response:

We fully agree with the reviewer that clearer sectioning and logical organization are critical for readability and scientific rigor. In response, we performed a major structural revision throughout the manuscript. Key changes include:

- **Reorganized the Methods section** into five distinct subheadings to separate the study context, analytical steps, and model definitions:
    - Study sites and data sources (Lines 104–125)
    - Forecasting workflow (Lines 126–131)
    - Time-series decomposition and seasonality testing (Lines 140–154)
    - Model descriptions (Lines 155–221)
    - Model evaluation and forecasting (Lines 222–230)
      This restructuring ensures that the methodological pipeline is no longer embedded in the data description and is instead fully detailed in its own subsection (Forecasting workflow, Fig 1).
- **Results section** has been revised for clarity and sequencing:
    - Descriptive and time-series components (Lines 233–267)
    - Model performance (Lines 272–280)
    - Workload forecasting (Lines 316–331)
- **Discussion section** was reorganized into five clearly titled sub-sections, reflecting the major thematic insights:
    - Hospital-specific caseload patterns (Lines 353–381)
    - Seasonal disease trends (Lines 382–422)
    - Model interpretability (Lines 423–437)
    - Forecast implications and systemic gaps (Lines 438–458)
    - Limitations and future directions (Lines 459–478)
- **Conclusion** was reframed to emphasize the practical implications of the forecasting framework and potential national-scale applications (Lines 480–503).

We believe these changes significantly enhance the manuscript's clarity and logical flow.

All sections were revised and removed the redundancy for improving clarification and precision. We believe the revised structure now facilitates significantly improved readability and comprehension.

Q4. Clarity of Writing:
The article would benefit from careful editing to improve clarity and precision. For example, it states in the Abstract that "six time-series forecasting models" were used, but these are not listed at the time of mention. Furthermore, the statement that "Exponential Smoothing (ETS) demonstrated the highest accuracy for NEI" in the abstract lacks context—highest compared to which models? Evaluation metrics such as MAE, RMSE, and MASE are used without being defined or briefly explained in the manuscript, which may confuse readers unfamiliar with these terms.

Response:

We appreciate the reviewer's suggestions regarding clarity and technical precision. In response, we undertook careful editing of the Abstract and Methods sections to address these points explicitly:

- All six forecasting models are now listed at the first mention in the Abstract to provide immediate clarity to readers:

"...six time-series forecasting models—Autoregressive Integrated Moving Average and its seasonal model (ARIMA/SARIMA), Exponential Smoothing (ETS), Trigonometric, Box-Cox ARMA Trend Seasonality (TBATS), Neural Network Time Series Regression (NNTR), Prophet, and Extreme Gradient Boosting (XGBoost)—to predict monthly workloads..." (Lines 24–27)

- We also revised the **Abstract** phrasing on performance:

"The ETS model achieved the highest accuracy at NEI, outperforming all other models by effectively capturing its stable and seasonal caseload patterns. In contrast, the NNTR model performed best at DLD, where it accommodated irregular fluctuations likely driven by external factors." (Lines 31–34)

- In the Model Evaluation section, we defined the evaluation metrics:

“Forecast accuracy was evaluated using three metrics: Mean Absolute Error (MAE), Root Mean Squared Error (RMSE), and Mean Absolute Scaled Error (MASE). MAE measures simple average errors; RMSE penalizes larger errors more significantly; MASE provides a scale-independent error measure, comparing model forecasts against a naïve benchmark.” (Lines 225–229)

These improvements should make the manuscript more accessible to a broader scientific readership without compromising technical rigor.

Conclusion:
Overall, the topic and dataset are promising and relevant. However, the manuscript requires substantial revisions in structure, clarity, and justification to reach its full potential.

Response:

We thank the reviewer for acknowledging the relevance and potential of the study. In response to your concerns, we have undertaken substantial revisions throughout the manuscript to improve:

- **Structure:** The manuscript was edited thoroughly, removed redundancy, and logically separated subsections in both Methods and Discussion (see Lines 104–231 and 339–479).
- **Clarity:** Key concepts, methods, and results have been rewritten for precision and accessibility. Evaluation metrics and model performance are now clearly explained and contextualized (Lines 104–332; 225-229).
- **Justification:** We strengthened the rationale for forecasting in low-volume wildlife hospitals, emphasizing resource-intensive care, conservation significance, and practical applications (Lines 35-36, 83–87, 95-99, 340-342, 348-350, 454–455, 482-484, and 491-496).

We believe these revisions substantially improve the manuscript’s readability, scientific rigor, and overall contribution to veterinary and conservation medicine.
